# Severe Lithium Toxicity due to the Use of Unauthorized Weight Loss Medication

**DOI:** 10.1111/bdi.70101

**Published:** 2026-02-26

**Authors:** Georgios Ydraios, Rayan Nasserdine, Emilie Duret, Stergios Tsartsalis, Hélène Richard‐Lepouriel

**Affiliations:** ^1^ Mood Disorder and Anxiety Unit, Psychiatric Specialties Service, Geneva University Hospita Geneva Switzerland; ^2^ Department of Psychiatry University of Geneva Geneva Switzerland

## 1 Clinical Presentation

A 39‐year‐old female patient, followed in outpatient care for bipolar I disorder, effectively treated with lithium since January 2022 (a dose of 18 mmol 2× per day and latest plasma concentrations around 0.85 mmol/L during the last months), and flurazepam (30 mg/j) for sleep onset difficulties, was referred to the emergency department for involuntary admission on August 13 by her psychiatrist for suspicion of a manic episode with mixed features.

Upon arrival at the psychiatric emergency department, the patient was confused, with disorganized thinking, disoriented as far as the situation, the place and the time, a marked psychomotor slowing, bilateral miosis, spontaneous tremors, jaw dysarthria and muscle rigidity. She reported taking lithium as prescribed (18 mmol 2×/day), flurazepam (30 mg per day), and a weight‐loss treatment which she had procured, without prescription, online (of a composition unknown to the patient). Biological tests revealed acute kidney injury (KDIGO III), with elevated lithium levels at 3.94 mmol/L, hypokalemia at 2.6 mmol/L, and hypophosphatemia at 0.22 mmol/L. A prolonged QTc of 570 ms was also noted. A brain CT scan ruled out any intracranial hemorrhage.

### 1.1 Initial Management

The patient was transferred to intensive care for a lithium poisoning‐associated toxic encephalopathy. All previously administered pharmacological treatment was suspended. Benzodiazepines (lorazepam 5 mg per day) were administered to manage the neurological symptoms with good effect. After two dialysis sessions, her condition improved, with a return to normal diuresis, creatinine levels within the normal range, and lithium levels below 1 mmol/L. Her clinical presentation improved as well. After her non‐psychiatric condition was stabilized, the patient was transferred to a specialized inpatient mood disorders unit of our Hospital. She was euthymic. Lithium treatment was reinstated upon arrival in our unit, given that the non‐psychiatric state had been stabilized and lithium serum concentration was below 1 mmol/L, to a final dose of 30 mmol/day, according to current clinical practice [1].

We conducted a toxicological screening using LC–MS/MS of the weight‐loss treatment, which revealed that it contained fluoxetine, furosemide, hydrochlorothiazide, metformin, and sibutramine. These two diuretics likely contributed to the elevated lithium levels and the acute kidney injury.

### 1.2 Psychiatric History

**FIGURE 1:**
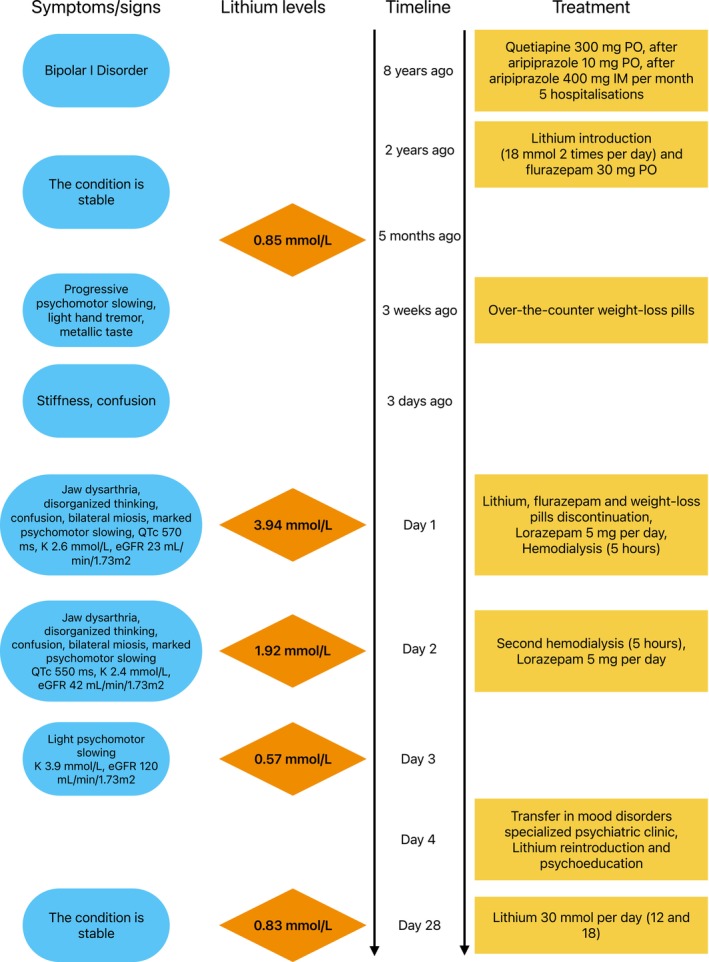
Life chart.

The patient has bipolar I disorder, diagnosed in 2016. She has had three psychiatric hospitalizations for manic episodes with behavioral disturbances, including hetero‐aggressive behavior and persecutory delusions and two for major depressive episodes. She has been under outpatient care with a history of variable therapeutic compliance. She had been consequently treated with quetiapine up to 300 mg per day, aripiprazole (per os) up to 10 mg per day and intramuscular long‐acting aripiprazole (400 mg per month). After two more hospital admissions for a manic episode following treatment discontinuation (the latest taking place in January 2022), lithium was introduced, and no new episode was reported since (Figure 1).

In the end of July 2024, the patient started taking over‐the‐counter weight‐loss pills ordered online from Brazil, without knowing their exact composition. According to her immediate family, since early August, she had experienced hand tremor, progressive psychomotor slowing and stiffness, culminating with a state of confusion over the last 3 days preceding her admission. She noted that she had developed tremors in her upper limbs, a metallic taste, and nausea after she started taking the weight‐loss pills. The use of these weight‐loss pills was not reported to her psychiatrist. Lithium treatment was continued as prescribed throughout the course of the weight‐loss use.

## 2 Discussion

Lithium remains a cornerstone in managing bipolar disorder due to its efficacy in stabilizing mood fluctuations. However, its narrow therapeutic index necessitates rigorous monitoring, as interactions with other substances can precipitate toxicity. This case exemplifies the dangers associated with unregulated weight‐loss supplements, which may contain undisclosed pharmacologically active ingredients [2]. The toxicological analysis of the patient's weight‐loss product revealed the presence of fluoxetine, furosemide, hydrochlorothiazide, metformin, and sibutramine. The inclusion of diuretics such as furosemide and hydrochlorothiazide is particularly concerning, as they can reduce renal lithium clearance, leading to elevated serum lithium levels and potential toxicity. Additionally, sibutramine, an appetite suppressant withdrawn from the market due to cardiovascular risks, and fluoxetine, a selective serotonin reuptake inhibitor, can further complicate the clinical picture through their own side effect profiles and potential interactions. The prevalence of dietary supplements adulterated with pharmaceutical agents is a growing public health concern. A study assessing the US Food and Drug Administration's Tainted Supplements Database from 2007 through 2016 identified numerous products marketed for weight loss containing undeclared substances, including sibutramine and various diuretics [3]. These adulterated supplements pose significant health risks, especially when consumed without medical supervision. According to research, nonprescription weight‐loss product use remains common, despite the potential for severe adverse effects [4]. Additionally, a systematic review identified a high prevalence of adolescent use of nonprescription weight‐loss products, further emphasizing the necessity of stricter regulations and public awareness campaigns [5].

Patients with bipolar disorder may seek over‐the‐counter weight‐loss products to address medication‐associated weight gain, often without disclosing this to their healthcare providers. This behavior underscores the necessity for clinicians to proactively inquire about all substances patients are taking, including non‐prescription products. Failure to do so can result in a confirmation bias, where new or worsening symptoms are misattributed solely to psychiatric causes, potentially overlooking underlying non‐psychiatric issues such as medication interactions or toxicities. Beyond the pharmacological interaction with diuretics, rapid weight loss and associated volume depletion may have further reduced renal lithium clearance, thereby promoting lithium accumulation. Psychoeducation plays a pivotal role in preventing such adverse events. Educating patients on the potential risks of unapproved supplements and the importance of reporting all ingested substances can aid in early identification of toxicity. Moreover, informing patients about the early signs of lithium overdose—including gastrointestinal disturbances, tremors, confusion, and neuromuscular excitability—can facilitate prompt medical attention and intervention.

In conclusion, this case highlights the imperative for comprehensive medication reconciliation and patient education in those receiving lithium therapy. Clinicians must maintain a high index of suspicion for potential interactions with over‐the‐counter products and provide thorough education on the risks associated with unregulated supplements and the early manifestations of lithium toxicity.

## 3 Learning Points

- This case underscores the critical need for comprehensive medication reconciliation in patients undergoing lithium therapy, including inquiry into non‐prescribed treatments and supplements.
- Psychoeducation on recognizing early symptoms of lithium toxicity is essential to prevent severe complications.

## Data Availability

The data that support the findings of this study are available from the corresponding author upon reasonable request.
